# Relational bullying and disordered eating: Testing a moderated mediation model of the role of shame and self-compassion

**DOI:** 10.3389/fpsyg.2023.968046

**Published:** 2023-04-05

**Authors:** Lindsay A. Bellows, Laura E. Couturier, Leigh C. Dunn, Jacqueline C. Carter

**Affiliations:** ^1^Department of Psychology, Memorial University of Newfoundland, St. John’s, NL, Canada; ^2^Faculty of Medicine, Memorial University of Newfoundland, St. John’s, NL, Canada

**Keywords:** eating disorders, disordered eating, social bullying, relational bullying, shame, self-compassion, young adults

## Abstract

**Objective:**

Experiences of relational bullying (RB) in adolescence are associated with the development of disordered eating. This association may be related to heightened shame resulting from perceived social inferiority, low social rank, and/or negative evaluation by others. Self-compassion may act as a protective factor against the influence of RB on shame and disordered eating. In the current study, we investigated whether shame mediated the relationship between recalled RB and current disordered eating in a sample of young adults. Then, using conditional process analysis, we examined whether the observed mediation was moderated by self-compassion.

**Method:**

Participants were 359 young adults (aged 17–25) who completed online self-report measures of recalled RB experiences and current disordered eating, shame, and self-compassion.

**Results:**

Experiences of RB were positively related to current shame and disordered eating, and negatively related to current self-compassion, with small-to-medium effect sizes. The association between RB and disordered eating was partially mediated by shame, and this mediation was moderated by self-compassion.

**Discussion:**

Our results suggest that young adults with lower self-compassion are more likely to demonstrate a relationship between recalled RB and disordered eating through the mechanism of shame. These findings have important implications for both anti-bullying awareness and eating disorder prevention programs.

## 1. Introduction

Bullying has been defined as repeated acts of physical, verbal, or indirect aggression deliberately intended to hurt someone in the context of a power imbalance (Olweus, 1993). Relational bullying (RB) is a form of indirect bullying that involves attempts to damage a person’s social relationships (e.g., social exclusion), social status (e.g., rumor-spreading, exposing secrets), or sense of social belonging (Crick and Grotpeter, 1995). RB has been shown to predict a variety of mental health concerns including body image disturbances and disordered eating behaviors (e.g., Lunde et al., 2006; Frank and Acle, 2014; Beekman et al., 2017). Despite these well-documented associations, RB is often viewed as a less serious form of bullying in comparison with physical and verbal bullying (Jacobsen and Bauman, 2007; Costley et al., 2014) and has received far less research attention.

Gilbert (2003) has argued that humans are innately motivated to seek social approval and belonging, and that shame may result from feeling that one is inferior to others in terms of social rank or attractiveness (Gilbert, 1997; Gilbert, 2000). RB is associated with a power/social rank differential between aggressor and victim (Volk et al., 2014) and is theorized to be perpetrated with the goal of social dominance (Vaillancourt et al., 2003; Olthof et al., 2011). Thus, the link between RB and disordered eating may be related to increased shame as a result of perceived social inferiority or low social rank. Shame is a known predictor of disordered eating (e.g., Kelly and Carter, 2012; Cavalera et al., 2016; Gois et al., 2018; Blythin et al., 2020) and has been associated with a fear of negative evaluation by others (Gilbert, 2000; Sznycer et al., 2016). Evidence for the interrelationships between perceived social rank, perceived attractiveness, shame, and disordered eating comes from research showing that disordered eating is related to feelings of social insecurity or inferiority (Ferreira et al., 2013a,b; Pinto-Gouveia et al., 2014) as well as shame (Mendes and Ferreira, 2020). Therefore, a model of disordered eating wherein low perceived social rank is associated with disordered eating among vulnerable individuals through increased shame may explain why shame has been found to mediate the relationship between bullying and disordered eating in adolescent females (Duarte et al., 2015, 2017). This model may also explain why both shame (i.e., the perception that others evaluate you negatively) and self-criticism have been found to mediate the association between lack of affiliative memories with peers and disordered eating (Mendes et al., 2017). To our knowledge, no studies to date have examined the role of shame in explaining the relationship between RB and disordered eating.

Self-compassion, an attitude of kindness and acceptance toward one’s personal distress and disappointments, is negatively related to shame (Johnson and O’Brien, 2013; Woods and Proeve, 2014). Individuals who have endured early experiences of abuse, criticism or neglect, tend to respond to distress with self-criticism rather than self-compassion (Gilbert, 2005). Relevant to the current study, higher self-compassion is associated with lower shame in those who have been bullied, and therefore it may act as a protective factor in the relationship between experiences of bullying and shame (Beduna and Perrone-McGovern, 2019). In addition, self-compassion is associated with reduced eating disorder psychopathology in both non-clinical and clinical samples (e.g., Wasylkiw et al., 2012; Ferreira et al., 2013c, 2019; Kelly et al., 2014; Turk and Waller, 2020). A closely related concept, self-reassurance, has also been found to be associated with less disordered eating and to be a mediator of the relationship between feeling accepted by others and disordered eating (Mendes et al., 2019). It has also been found that self-reassurance interacts with bullying to predict disordered eating in adolescent females, such that those who had experienced bullying but had high self-reassurance reported less disordered eating behavior than those with low self-reassurance (Duarte and Pinto-Gouveia, 2017). Taken together, these findings suggest that self-compassion may be a protective factor against the development of shame and disordered eating among young people who have experienced RB.

The aim of the current study was to investigate the association between recalled RB and current disordered eating in a young adult sample. Specifically, we examined whether RB predicted disordered eating and whether this relationship was mediated by shame. In addition, we performed a moderated mediation analysis to evaluate whether this mediation was moderated by self-compassion.

To our knowledge, this is the first study to investigate the mediating role of shame and the moderating role of self-compassion in the relationship between past RB and current disordered eating among young adults using moderated mediation analysis. Our study also attempted to address certain methodological shortcomings of previous studies. First, while previous research has frequently excluded males, this study included individuals of all genders. Second, while previous research has been largely limited to either clinical samples or young adolescent samples, the current study focused on young adults. Finally, the present study focused specifically on RB since little is known about whether this type of bullying is associated with disordered eating.

## 2. Materials and methods

This study employed a cross-sectional design. All aspects of the study were approved by the Interdisciplinary Committee on Ethics in Human Research (ICEHR) at Memorial University of Newfoundland.

### 2.1. Participants

Participants were recruited from undergraduate psychology classes where students participated for course credit, as well as through advertisements posted on social media platforms (e.g., Facebook and Instagram) and around the university campus. In total, 390 participants completed the study measures. Of these, 31 participants did not meet the age criteria, did not provide informed consent, or did not agree to have their data included in the study. Therefore, the final sample consisted of 359 young adults between the ages of 17 and 25 years. Participants consisted of 307 females (85.8%), 46 males (12.8%), and 5 transgender/non-binary (1.4%) individuals. The mean age of the sample was 20 years (*SD* = 1.8). Self-reported body mass index (BMI) ranged from 15.1 to 51.7 (*M* = 24.8, *SD* = 5.3). Participants were 89.1% Caucasian/White and 46.5% were single. Of the 359 participants, 296 (82.5%) reported they had never been diagnosed with an eating disorder, 28 (7.8%) reported they had been diagnosed with an eating disorder, and 35 (9.7%) reported that they believed they currently had an eating disorder.

### 2.2. Measures

#### 2.2.1. Bullying

The Forms of Bullying Scale - Victimization (FBS-V; Shaw et al., 2013) was used to measure the amount and type of RB participants recalled experiencing either offline or online. The FBS-V is a 10-item self-report questionnaire that measures the frequency of five types of bullying experiences (verbal, threatening, physical, social, relational) using a 5-point Likert scale ranging from 1 (*this did not happen to me*) to 5 (*several times a week or more*). The mean score ranges from 1 to 5 with higher scores indicating more frequent bullying. The FBS-V has been shown to have good construct validity, convergent validity, and discriminant validity (Shaw et al., 2013). Participants were asked to think back as far as the beginning of junior high school (approximately age 13 and older) when responding. Given that social (e.g., “Secrets were told about me to others to hurt me”) and relational bullying (e.g., “I was hurt by someone trying to break up a friendship”) are both forms of indirect bullying (Archer and Coyne, 2005; Heilbron and Prinstein, 2008) and these two subscales were highly correlated (*r* = 0.744), they were combined to form one subscale in the current study (RB). McDonald’s ω hierarchical for the social and relational bullying scales combined was 0.85, and McDonald’s ω total for the combined scale was 0.91.

#### 2.2.2. Disordered eating

The Eating Disorder Examination Questionnaire 6.0 (EDE-Q; Fairburn and Beglin, 2008) Global Score was used to measure disordered eating. The EDE-Q is a 36-item self-report questionnaire that assesses the severity of Eating Concern (e.g., “Have you had a definite fear of losing control over eating?), Dietary Restraint (e.g., “Have you been deliberately trying to limit the amount of food you eat to influence your shape or weight?), Shape Concern (e.g., “Have you had a definite desire to have a totally flat stomach?”) and Weight Concern (e.g., “Have you had a definite fear that you might gain weight?) over the past 28 days. Items are rated on a 7-point scale. Global scores range from 0 to 6 with higher scores indicating more severe symptoms. The reliability and validity of the EDE-Q 6.0 have been well established (Berg et al., 2012). In the current study, McDonald’s ω hierarchical for the EDEQ global was 0.78, and McDonald’s ω total for the global scale was 0.97.

#### 2.2.3. Self-compassion

The Self-Compassion Scale – Long Form (SCS; Neff, 2003) was used to measure self-compassion. The SCS is a 26-item self-report questionnaire that measures the degree to which participants respond to feelings of inadequacy or emotional pain with self-kindness versus judgment (e.g., “I try to be loving toward myself when I’m feeling emotional pain”), common humanity versus isolation (e.g., “When things are going badly for me, I see the difficulties as part of life that everyone goes through”), and mindfulness versus overidentification (e.g., “When I’m feeling down I try to approach my feelings with curiosity and openness”). The questionnaire uses a 5-point Likert scale from 1 *(almost never)* to 5 (*almost always*). The total mean score can range from 1 to 5 with higher scores indicating higher levels of self-compassion. The SCS has been shown to have good internal consistency (Allen et al., 2012; Neff and Pommier, 2013), construct validity (Neff, 2003; Neff and Pommier, 2013), and discriminant validity (Neff, 2003; Neff and Vonk, 2009). McDonald’s ω hierarchical in the current study was 0.74, and McDonald’s ω total was 0.96.

#### 2.2.4. Experiences of shame

The Experience of Shame Scale (ESS; Andrews et al., 2002) is a 25-item self-report measure of feelings of shame about one’s character, behavior or body over the past year. The ESS consists of three factors: shame about one’s character (e.g., “Have you felt ashamed of the sort of person you are?”), shame toward one’s behavior (e.g., “Have you tried to cover up or conceal any of your personal habits?”), and shame about one’s body (e.g., “Have you worried about what other people think of your appearance?”). Items are rated using a scale ranging from 1 (*not at all*) to 4 (*very much*). The total score can range from 25 to 100 with higher scores indicating higher levels of shame. The ESS has acceptable internal consistency, test–retest reliability, and construct validity in university students (Andrews et al., 2002). McDonald’s ω hierarchical for the ESS was 0.75, and McDonald’s ω total for the scale was 0.96.

### 2.3. Procedure

Participants accessed the study by clicking or typing out the anonymous link given (or using a QR code to access the website) which directed them to the survey platform Qualtrics. Upon entering the webpage for the study, participants were provided with an informed consent form, study measures, and debriefing form. The first two measures (SCS, and ESS) were presented in random order to prevent any order effects. Next, participants completed the EDE-Q and FBS in that order. The FBS was presented last to prevent any priming effects of recalled bullying experiences on responses to the other scales. Finally, participants were directed to a debriefing form and were given the option to enter their e-mail in a draw to win one of two $50 gift cards.

### 2.4. Statistical analyses

**Figure 1:**
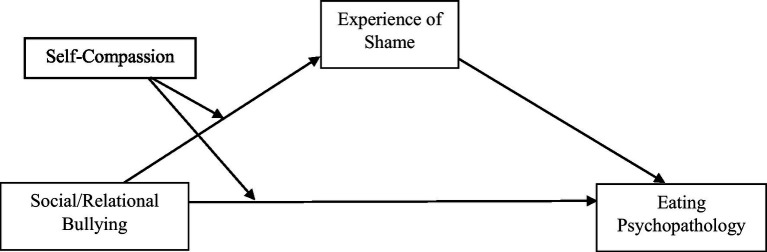
Conceptual model diagram of conditional process analysis.

Statistical analyses were completed using SPSS Version 28.0 (IBM Corp, 2021). First, the data were screened to ensure regression assumptions were met. McDonald’s ω hierarchical and McDonald’s ω total were calculated for each scale using the *psych* package in RStudio (Revelle, 2022). To avoid multicollinearity issues, predictor variables were centered prior to analyses. First, zero-order Pearson correlations were generated to examine the relationships between all study variables. Next, to examine whether shame mediated the relationship between recalled RB and disordered eating, a mediation analysis using ordinary least squares path analysis was conducted using the PROCESS Macro for SPSS (Model 4; Hayes, 2013) with BMI entered as a covariate. The bootstrap method was used to evaluate the significance of the mediation. Finally, to determine whether the observed mediation was moderated by self-compassion, a conditional process analysis was conducted using the PROCESS Macro for SPSS (Model 8; Hayes, 2013) with BMI entered as a covariate. A diagram of the conceptual model for the conditional process analysis is provided in Figure 1.

## 3. Results

### 3.1. Data screening

Data were inspected for violations of regression assumptions (i.e., independence of observations, linearity of relationships among variables, homoscedasticity of error values, lack of multicollinearity among independent variables, and normally distributed error values; Hayes, 2018). To verify that these assumptions were met, a one-step procedure outlined by Clement and Bradley-Garcia (2022) was conducted, with EDEQ scores entered as a dependent variable and ESS, SCS and FBSV scores entered as predictors. Results revealed that the assumption of independence of residuals was met (Durbin-Watson statistic = 2.057).

Visual inspection of a scatterplot of studentized residuals and unstandardized predicted values revealed that the assumption of linearity was met as the data scattered in a horizontal manner suggesting that the relationships between all variables in the model are linear. Further inspection of this scatterplot confirmed that the residuals fit a rectangular shape. Thus, the error values were randomly scattered across different values of the dependent variable (EDEQ) and the assumption of homoscedasticity was met. The VIF statistics for each of the predictor variables revealed that there was also a lack of multicollinearity (VIF(ESS) = 1.865, VIF(SCS) = 1.787, VIF(FBS) = 1.151). Lastly, the residuals appeared slightly positively skewed based on the scatterplot and lack of normality was confirmed by the Normal Probability Plot (P–P Plot), with slight deviation from the diagonal line. However, since regression is robust against non-severe violations of normality (Hayes, 2018), data were not transformed beyond mean centering. Missing data were imputed with the mean value of the scale/subscale for participants who completed at least 90% of assessment measure.

### 3.2. Preliminary analyses

The mean, standard deviation and range for each of the study measures are presented in Table 1. Table 2 presents a Pearson bivariate correlation matrix for the relationships between the study variables. Recalled RB scores were significantly positively associated with disordered eating (*r*(357) = 0.38, *p* < 0.001)and shame (*r*(357) = 0.35, *p* < 0.001), and significantly negatively associated with self-compassion (*r*(357) = −0.29, *p* < 0.001), with medium effect sizes. Recalled RB scores significantly predicted disordered eating when BMI was controlled, t(356) = 7.49, *p* < 0.001, change in *R*^2^ = 0.12, with a medium effect size.

**Table 1:** Descriptive statistics for study measures.

Measure	Mean	SD	Min.	Max.	Range
FBS	1.164	1.013	0.000	4.000	4.000
EDE-Q	1.776	1.291	0.000	5.175	5.175
SCS	2.723	0.721	1.033	4.833	3.800
ESS	66.009	16.541	28.000	100.000	72.000

FBS, Forms of Bullying Scale, ESS, Experience of Shame Scale, SCS, Self-Compassion Scale, and EDEQ, Eating Disorder Examination Questionnaire 6.0.

**Table 2:** Correlations between study variables.

Variable	1	2	3	4
1. Relational bullying	—			
2. Disordered eating	0.38*	—		
3. Self-compassion	−0.29*	−0.52*	—	
4. Experiences of shame	0.35*	0.60*	−0.66*	—

*p < 0.001.

### 3.3. Shame mediates the relationship between recalled RB and disordered eating

**Figure 2:**
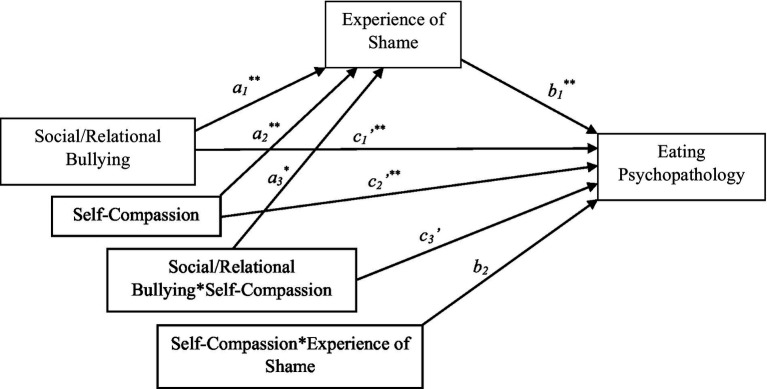
Statistical model of conditional mediation. See Supplementary Table S1 in Supplementary File for model coefficients. *p* < 0.005, ^**^p < 0.001.

As expected, shame partially mediated the relationship between recalled RB and disordered eating while controlling for BMI. As displayed in Figure 2, participants with higher RB scores had higher shame scores (*a* = 5.758, *SE* = 0.808, *p* < 0.001) and participants with higher shame scores had higher disordered eating scores (*b* = 0.041, *SE* = 0.004, p < 0.001). A bootstrap confidence interval based on 5,000 bootstrap samples was above zero (0.167 to 0.311) for the indirect effect of RB on disordered eating as mediated through shame (*a* x *b* = 0.237; SEa x b = 0.037). The direct effect of RB on disordered eating was also significant (*c*’ = 0.245; SEc = 0.062, p < 0.001) suggesting a partial mediation by shame. The bootstrap confidence interval based on 5,000 bootstrap samples remained above zero (0.152 to 0.292) for the indirect effect of RB on disordered eating as mediated through shame (*a* x *b* = 0.218; SEa x b = 0.036). The direct effect of RB on disordered eating also remained significant (*c*’ = 0.233; SEc = 0.055, p < 0.001) suggesting a partial mediation even when BMI is entered into the model.

### 3.4. Moderated mediation model with shame as mediator and self-compassion as moderator

As outlined in Figure 1, the conditional indirect effect of self-compassion on the relationship between RB and disordered eating *via* shame (i.e., shame) was tested (while controlling for BMI). Results indicated that the interaction between self-compassion and RB significantly predicted levels of the mediator, shame (*a*_3_ = 2.682, *SE* = 0.940, *p* < 0.001). In addition, self-compassion was a significant moderator of the direct relationship between recalled RB and disordered eating (*c*_3_ = −0.202, *SE* = 0.076, *p* < 0.01). See Figure 2 and associated model coefficients in Supplementary Table S1 (see Supplementary materials). The overall moderated mediation model was supported with the index of moderated mediation = 0.839 (95% CI = 0.0315; 0.1507). As zero did not fall within the upper and lower CIs, this indicates that the mediation was moderated by self-compassion. The conditional indirect effect was weakest in those high in self-compassion (1 SD above the mean of SCS; effect = 0.013, SE = 0.093, 95% CI = −0.170; 0.197) and strongest in those low in self-compassion (1 SD below the mean of SCS, effect = 0.305, SE = 0.063, 95% CI = 0.181; 0.428), suggesting that individuals who are low in self-compassion are more likely to demonstrate a relationship between recalled RB and disordered eating through the mechanism of shame compared to individuals who are high in self-compassion.

## 4. Discussion

This study was the first to our knowledge to test a moderated mediation model of the role of shame and self-compassion in the relationship between recalled experiences of RB and current disordered eating among young adults. As expected, it was found that RB predicted disordered eating and that this relationship was partially mediated by shame, after accounting for BMI. This suggests that feelings of shame may contribute to the development of disordered eating among those who recall experiencing RB. Further, self-compassion moderated this mediation. This implies that relatively lower self-compassion was associated with a stronger relationship between recalled RB and current disordered eating through the mechanism of increased shame. Conversely, those who recalled experiencing RB but had relatively higher self-compassion reported lower risk disordered eating *via* shame. Our findings support prior research suggesting that bullying is associated with disordered eating and that shame may explain this relationship. We also found evidence that self-compassion may buffer the relationship between RB and disordered eating through shame. These results have both theoretical and clinical implications.

### 4.1. The role of shame

Shame was found to partially account for the relationship between RB and disordered eating. This provides support for Gilbert’s (2005) theory that negative experiences such as bullying tends to produce increased shame which increases the risk of psychopathology including disorder eating. Our results are also consistent with previous findings suggesting that shame mediates the relationship between global bullying and disordered eating in clinical samples (Sweetingham and Waller, 2008) and that body image shame mediates this relationship in nonclinical samples (Duarte et al., 2015, 2017). One possible explanation as to why the current study found shame to only partially mediate the relationship between RB and disordered eating in contrast to the full mediation found in previous studies is that different measures of both bullying and shame were used. Previous studies investigated global bullying instead of RB and body image shame rather than global shame. Therefore, it is possible that increased body image shame may be a stronger mediator of the relationship between bullying and disordered eating. In addition, other potential mediators of the relationship between RB and disordered eating may account for why only a partial mediation was found. For example, previous research has found socially prescribed perfectionism to be associated with bullying victimization (Haltigan and Vaillancourt, 2018), as well as to mediate links between social variables, such as parenting, and the development of disordered eating (Sassaroli et al., 2011). Given this, it is possible that socially prescribed perfectionism may also act as a partial mediator in the relationship between RB and disordered eating.

### 4.2. The role of self-compassion

In support of previous findings in clinical and young adolescent samples (e.g., Beekman et al., 2017; Duarte and Pinto-Gouveia, 2017), self-compassion significantly moderated the relationship between RB to disordered eating (as mediated by shame) after accounting for BMI. This is in line with Gilbert’s (2005, 2011) theory that individuals with low self-compassion have difficulty accessing soothing emotions, such as warmth and reassurance, when faced with difficult experiences such as RB. Consequently, they tend to respond with self-criticism and shame which can trigger maladaptive coping responses such as disordered eating. Our findings are also consistent with growing evidence that self-compassion interventions may be helpful in reducing disordered eating and suggest that reduced shame may be the mechanism through which this occurs (Ferreiri et al., 2019; Turk and Waller, 2020). Our results are also consistent with previous findings showing that higher self-compassion is associated with lower shame among those who have been bullied (Beduna and Perrone-McGovern, 2019), suggesting that self-compassion may act as a protective factor in the relationship between experiences of bullying and disordered eating by reducing feelings of shame.

### 4.3. Strengths, limitations, and future directions

The present study had a number of strengths. First, we focused on RB rather than global bullying since little is currently known about the relationship between indirect forms of bullying and disordered eating. Second, we recruited a nonclinical sample of young adults of various genders since previous research has mainly focused on clinical adolescent samples. In addition, we used well-validated and widely use measures.

The current study also had a number of limitations. First, given the cross-sectional nature of the data, the present mediation analysis was an *atemporal* mediation (i.e., as opposed to a *temporal* mediation, which requires longitudinal data) yielding correlational results (Winer et al., 2016). Consequently, it is not possible to determine the temporal relationships between the variables that we studied, nor is it possible to draw causal conclusions. Second, there may be limitations regarding the generalizability of the study sample given that the participants were predominantly white female students. Other possible limitations to note include the potential for self-selection bias given that recruitment advertisements included the words “peer experiences” and “eating behavior,” and the potential for memory inaccuracies given that young adult participants were asked to retrospectively recall bullying experiences as far back as junior high school. Also of note, the FBS-V was standardized on 12-15-year-old individuals but has been used with adult samples (e.g., Semenyna and Vasey, 2015; Ratcliff et al., 2020). Future directions for this line of research include longitudinal studies to investigate the relationship between RB and disordered eating using more diverse community samples. It may also be pertinent to examine these interrelationships in men as well as gender-diverse individuals. In addition, our findings suggest that RB is a serious form of bullying which should be targeted in anti-bullying campaigns and potentially in eating disorder prevention programs. One possible approach to the prevention of disordered eating may be promoting the development of self-compassion among victims of RB.

## 5. Conclusion

The findings of this study have potentially important implications for both clinical and school settings. First, while further research is needed, our results suggest that RB is a serious form of bullying that is associated with negative mental health outcomes among young people, including disordered eating. Second, the findings point to the importance of shame and self-compassion in the relationship between RB and disordered eating, suggesting that interventions aimed at reducing shame through the development of self-compassion among RB victims may decrease the risk for disordered eating.

## Data availability statement

The raw data supporting the conclusions of this article will be made available by the authors, without undue reservation.

## Ethics statement

The studies involving human participants were reviewed and approved by Interdisciplinary Committee on Ethics in Human Research (ICEHR) at Memorial University of Newfoundland. The patients/participants provided their written informed consent to participate in this study.

## Author contributions

LB and LC contributed to conceptualization, methodology, investigation, data analysis, and writing the manuscript. LD contributed to writing the manuscript and preparing it for publication. JC contributed to conceptualization, methodology, supervision, and manuscript writing. All authors contributed to the article and approved the submitted version.

## Funding

LB was supported by a scholarship from the Social Sciences and Humanities Research Council (SSHRC). LB, LC, and LD received trainee awards from the Janeway Research Foundation. LC is supported by a scholarship from the Canadian Institutes of Health Research (CIHR).

## Conflict of interest

The authors declare that the research was conducted in the absence of any commercial or financial relationships that could be construed as a potential conflict of interest.

## Publisher’s note

All claims expressed in this article are solely those of the authors and do not necessarily represent those of their affiliated organizations, or those of the publisher, the editors and the reviewers. Any product that may be evaluated in this article, or claim that may be made by its manufacturer, is not guaranteed or endorsed by the publisher.
