# Supplementary material for: Relational bullying and disordered eating: Testing a moderated mediation model of the role of shame and self-compassion
Source: Front Psychol. 2023 Apr 5;14:968046. doi: 10.3389/fpsyg.2023.968046 (PMC10114926; doi:10.3389/fpsyg.2023.968046)
Supplement: Supplementary file 1 [file Table_1.docx]

**Supplementary Table 1.** *Model Coefficients for the Conditional Process Model*

|  | Consequent | | | | | | | |
| --- | --- | --- | --- | --- | --- | --- | --- | --- |
|  |  | ESS | | |  | EDE-Q | | |
| Antecedent | Label | Coefficient | *SE* | *p* | Label | Coefficient | *SE* | *p* |
| FBS | *a_1_* | 3.54 | 0.94 | < .001 | *c_1_’* | 0.29 | 0.06 | < .001 |
| ESS |  | — | — | — | *b_1_* | 0.03 | 0.00 | < .001 |
| SCS | *a_2_* | -13.62 | 0.93 | < .001 | *c_2_’* | -0.81 | 0.08 | < .001 |
| FBS*SCS | *a_3_* | 2.69 | 0.94 | < .005 | *c_3_’* | -0.12 | 0.08 | .090 |
| SCS*ESS |  | — | — | — | *b_2_* | -0.01 | 0.01 | .118 |
| constant | *i_M_* | 108.07 | 3.87 | < .001 | *i_y_* | -1.308 | 0.22 | .136 |
|  |  | *R*^2^ = 0.476  *F*(3,355) = 107.49 | | |  | *R*^2^ = 0.425  *F*(5,353) = 52.116 | | |

*Note.* FBS = Forms of Bullying Scale, ESS = Experience of Shame Scale, SCS = Self-Compassion Scale, and EDE-Q = Eating Disorder Examination Questionnaire 6.0 Global Score.
